# The SWI/SNF ATPase BRG1 facilitates multiple pro-tumorigenic gene expression programs in *SMARCB1*-deficient cancer cells

**DOI:** 10.1038/s41389-022-00406-6

**Published:** 2022-06-01

**Authors:** Kylie C. Moe, Jack N. Maxwell, Jing Wang, Cheyenne A. Jones, Grace T. Csaki, Andrea C. Florian, Alexander S. Romer, Daniel L. Bryant, Anthony L. Farone, Qi Liu, William P. Tansey, April M. Weissmiller

**Affiliations:** 1 Department of Biology, Middle Tennessee State University, Murfreesboro, TN 32132 USA; 2 Center for Quantitative Sciences, Vanderbilt University Medical Center, Nashville, TN 37240 USA; 3 Department of Biostatistics, Vanderbilt University Medical Center, Nashville, TN 37240 USA; 4 Department of Cell and Developmental Biology, Vanderbilt University School of Medicine, Nashville, TN 37240 USA; 5 Department of Biochemistry, Vanderbilt University School of Medicine, Nashville, TN 37240 USA

**Keywords:** Oncogenes, Paediatric cancer, Cancer genomics

## Abstract

Malignant rhabdoid tumor (MRT) is driven by the loss of the SNF5 subunit of the SWI/SNF chromatin remodeling complex and then thought to be maintained by residual SWI/SNF (rSWI/SNF) complexes that remain present in the absence of SNF5. rSWI/SNF subunits colocalize extensively on chromatin with the transcription factor MYC, an oncogene identified as a novel driver of MRT. Currently, the role of rSWI/SNF in modulating MYC activity has neither been delineated nor has a direct link between rSWI/SNF and other oncogenes been uncovered. Here, we expose the connection between rSWI/SNF and oncogenic processes using a well-characterized chemical degrader to deplete the SWI/SNF ATPase, BRG1. Using a combination of gene expression and chromatin accessibility assays we show that rSWI/SNF complexes facilitate MYC target gene expression. We also find that rSWI/SNF maintains open chromatin at sites associated with hallmark cancer genes linked to the AP-1 transcription factor, suggesting that AP-1 may drive oncogenesis in MRT. Interestingly, changes in MYC target gene expression are not overtly connected to the chromatin remodeling function of rSWI/SNF, revealing multiple mechanisms used by rSWI/SNF to control transcription. This work provides an understanding of how residual SWI/SNF complexes may converge on multiple oncogenic processes when normal SWI/SNF function is impaired.

## Introduction

Malignant rhabdoid tumor (MRT) and atypical teratoid/rhabdoid tumors (AT/RT) are rare and aggressive childhood cancers with almost all cases diagnosed in young children. Despite efforts to identify new strategies to treat rhabdoid tumors, survival rates remain poor and very few treatment options currently exist [1]. Almost 100% of rhabdoid tumors are defined by loss of *SMARCB1* (SWI/SNF Related, Matrix Associated, Actin Dependent Regulator of Chromatin, Subfamily B, Member 1), a tumor suppressor gene that encodes the SNF5 subunit of the SWI/SNF (Switch/Sucrose Non-Fermentable) chromatin remodeling complex. Loss of *SMARCB1* is the most common recurring mutation in these cancers [2], highlighting the extensive role that SNF5 plays in tumor suppression. Many of these roles have been clearly defined using the reintroduction of SNF5 into MRT cell lines as the model system. These include the ability of SNF5 to activate enhancers linked to cell differentiation and development genes [3], oppose repression of bivalent promoters that are also linked to cell development genes [4], compete with the non-canonical SWI/SNF subunit, BRD9 (Bromodomain Containing 9), for access to pan-SWI/SNF subunits [5, 6], and impede the transcriptional potential of the oncoprotein transcription factor MYC [7]. The breadth of cellular processes discovered to be regulated by SNF5 continues to explain how the loss of this tumor-suppressive subunit acts as a major insult to normal cellular function and creates the potential for cancer.

Three different SWI/SNF complexes—canonical BAF (cBAF, BAF stands for BRG1/BRM-Associated Factor), polybromo-associated BAF (pBAF), and non-canonical BAF (ncBAF, also known as GBAF [8])—have been characterized in detail over the past several years [9]. While each complex shares some pan-subunits, such as the ATPase subunit, BRG1 (Brahma-related gene-1) or BRM (Brahma), and the subunit BAF155, each complex is marked by several unique subunits and each shows distinct genomic localization patterns important for their proposed specific functions [9]. Of all three complexes, SNF5 is found in cBAF and pBAF complexes and upon SNF5 loss the remaining SWI/SNF subunits in these two complexes persist, along with ncBAF. Evidence for the maintenance of rhabdoid tumors requiring the involvement of the SWI/SNF complexes that are retained in the absence of SNF5 (called “rSWI/SNF” herein) [5, 6, 10] is compelling. Early studies to knock down the SWI/SNF ATPase BRG1 revealed that BRG1 is required for cell proliferation and tumor formation in the absence of SNF5 [10]. In addition, genomic approaches have shown that rSWI/SNF subunits such as BRG1 are localized to super-enhancers in MRT to control tumor cell survival [3]. More recently, the BRD9 subunit of the ncBAF complex was identified as a synthetic lethal target in SNF5-deficient cancers and was found to maintain gene expression in these cancers [5, 6]. Together, these data point to an essential role for rSWI/SNF in the maintenance of an oncogenic state, which is not unusual among cancers that show SWI/SNF subunit alteration [11].

Presumably, rSWI/SNF complexes should converge on known oncogenic pathways, but a direct connection between rSWI/SNF and specific oncogenes in MRT is lacking. Recently, we found that a major oncogene in cancer, MYC, becomes deregulated in MRT through loss of SNF5 [7], a finding that explains the activation of MYC target gene signatures observed in *SMARCB1*-deficient cancers [12–14]. We also discovered that rSWI/SNF subunits can interact physically and colocalize on chromatin with MYC [15], suggesting that rSWI/SNF can facilitate MYC oncogenic function in the absence of SNF5. Unfortunately, the influence that rSWI/SNF has on a particular oncogene like MYC has not been delineated, and while rSWI/SNF should facilitate tumor-relevant transcriptional programs through impacting oncogenic pathways, those specific pathways—and the mechanisms by which they are regulated—remain unclear.

In this present study, we sought to determine the impact of rSWI/SNF on MYC function and expose any mechanisms by which these complexes function using a selective and specific chemical degrader targeted against the SWI/SNF ATPase, BRG1 [16–18]. Depletion of BRG1 followed by transcriptome analysis shows that expression of MYC target genes is impaired, an effect that can occur rapidly following BRG1 loss. Analysis of how the chromatin remodeling activity of rSWI/SNF impacts the MRT transcriptome reveals that SWI/SNF complexes can regulate the open chromatin state at sites linked to multiple cancer hallmark genes, such as signaling, angiogenesis, and migration. Interestingly, sites where inhibition of rSWI/SNF reduces chromatin accessibility are enriched with AP-1 (Activator protein 1) transcription factor binding motifs and show a corresponding change in gene expression. However, overt changes to the open chromatin state cannot explain impaired MYC target gene expression, indicating that rSWI/SNF complexes may control MYC target gene expression through a mechanism that is not entirely dependent on chromatin remodeling. Therefore, our data reveal that rSWI/SNF complexes can drive oncogenic transcriptional programs in MRT through multiple mechanisms, only some of which are due directly to the function of SWI/SNF as a chromatin remodeling complex. This study informs how retained SWI/SNF complexes in MRT can converge on multiple oncogenic processes through diverse mechanisms following SNF5 loss and may predict specific oncogenic pathways that become activated across other cancers showing SWI/SNF subunit alteration and mutations.

## Results

### ACBI1 treatment causes acute degradation of BRG1

**Fig. 1:**
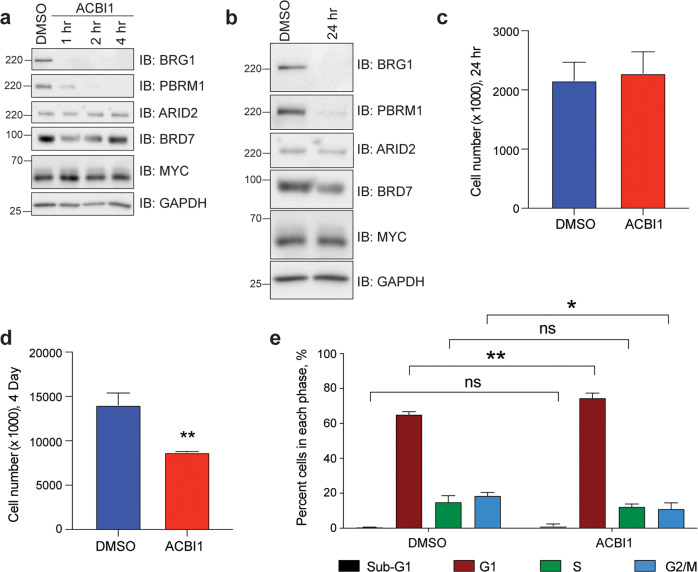
ACBI1 depletes SWI/SNF subunits BRG1 and PBRM1. **a** G401 cells were treated with 250 nM ACBI1 for the indicated times and protein lysates were probed for expression of BRG1, pBAF-specific subunits PBRM1, ARID2, and BRD7, and MYC. DMSO control is matched to longest treatment. GAPDH is used as a loading control. **b** G401 cells were treated as in **a** for 24 h and protein lysates probed for the indicated proteins. GAPDH is used as a loading control. **c** G401 cells were plated with 250 nM ACBI1 or DMSO control and cell growth monitored over 24 h (*n* = 5 biological replicates, error bars are standard error). **d** G401 cells in **c** were allowed to grow for 4 days in the presence of ACBI1 or DMSO control and cells counted at this timepoint (*n* = 5 biological replicates, error bars are standard error, **P = 0.004 using unpaired *t*-test, two-tailed between DMSO and ACBI1). **e** Cell cycle analysis of G401 cells treated with 250 nM ACBI1 or DMSO control for 4 days as determined by flow cytometry (*n* = 4 biological replicates, error bars are standard error, **P = 0.0015, *P = 0.0117 using unpaired *t*-test, two-tailed).

To broadly affect SWI/SNF function in MRT we treated the G401 cell line with commercially available proteolysis targeting chimera (PROTAC) degrader, ACBI1 [16–18] that targets the SWI/SNF ATPases, BRG1 and BRM, as well as the pBAF subunit, PBRM1 (polybromo 1). Treatment with 250 nM ACBI1 leads to a rapid reduction in levels of BRG1 and PBRM1 as early as 1 h following treatment, while not affecting protein levels of MYC (Fig. 1a). Cells treated for 24 h show similar specificity for BRG1 and PBRM1, with modest decreases in protein levels of other pBAF-specific subunits such as ARID2 (AT-Rich Interaction Domain 2) and BRD7 (Bromodomain Containing 7) (Fig. 1b). Despite rapid removal of BRG1 and PBRM1, G401 cells do not show a cell growth phenotype at 24 h (Fig. 1c), but by day 4 cell growth is impaired (Fig. 1d) and cell cycle analysis shows a significant increase of cells within the G1-phase (Fig. 1e). These data indicate that rSWI/SNF complexes are important for maintaining cellular function in MRT and are consistent with previous data revealing that rSWI/SNF subunits show synthetic lethality in SNF5-deficient cancers [5, 6, 10].

### Loss of SWI/SNF function results in diverse gene expression changes

**Fig. 2:**
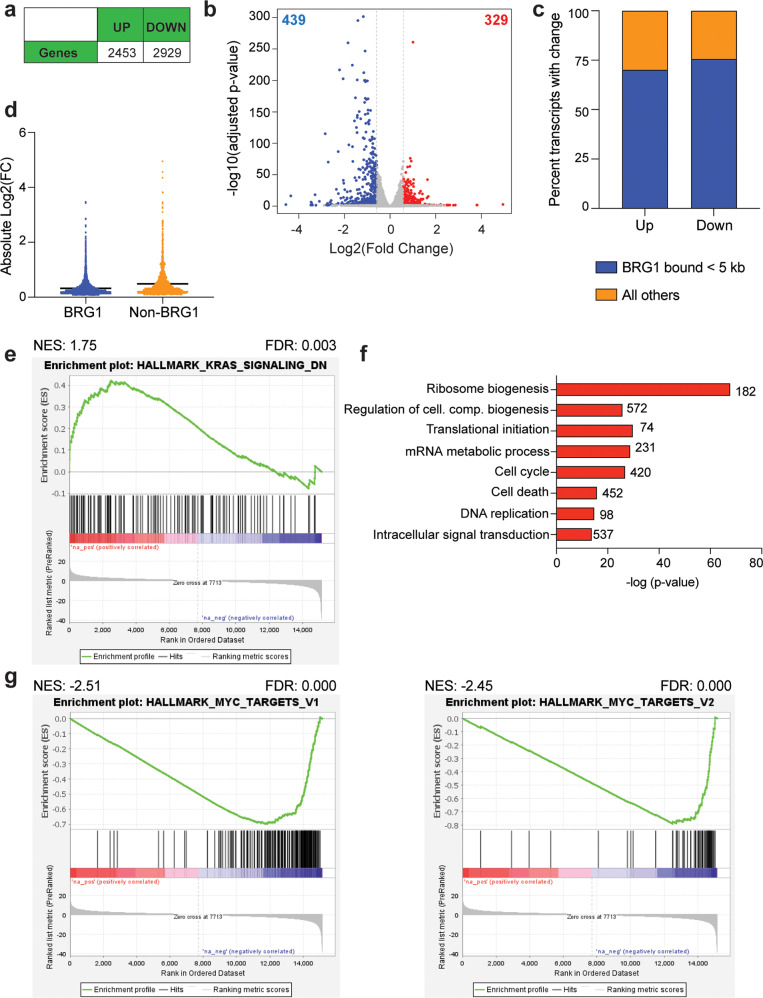
ACBI1 treatment causes diverse gene expression changes predominantly at BRG1-bound genes. **a** Total number of differentially expressed genes that were increased in expression (“up”) or decreased (“down”) in response to 24 h ACBI1 treatment. FDR < 0.05 was used for determining significantly changed genes. **b** Volcano plot showing all gene expression changes in each direction. Red represents genes that were increased in expression with a fold change > 1.5 and blue represents genes that were decreased in expression with a fold change < −1.5. **c** Transcripts with a change in each direction were compared to publicly available BRG1 ChIP-seq data (GSE90634, [4, 15]). Regardless of direction, most transcripts that significantly change show BRG1 binding within 5 kb of the transcription start site. **d** Scatter dot plot showing the absolute value of all individual log2FC changes for genes bound by BRG1 versus non-bound as defined in **c**. Line falls at mean of each sample. **e** Gene set enrichment analysis (GSEA) for all gene expression changes compared against MSigDB hallmark datasets. Transcripts changed following ACBI1 treatment show positive enrichment within ranked list of genes in the “KRAS signaling down” hallmark dataset. **f** Gene ontology (GO) analysis of transcripts that are decreased by ACBI1 treatment. Number of genes in each term are shown next to the bar, cell. com. = cellular component. **g** GSEA analysis showing the two most significant MSigDB hallmark lists that resulted from comparing all gene expression changes with ACBI1 treatment against the MSigDB hallmark datasets. Transcripts that are changed following ACBI1 treatment show a negative enrichment within a ranked list of MYC targets, V1 and V2.

**Fig. 3:**
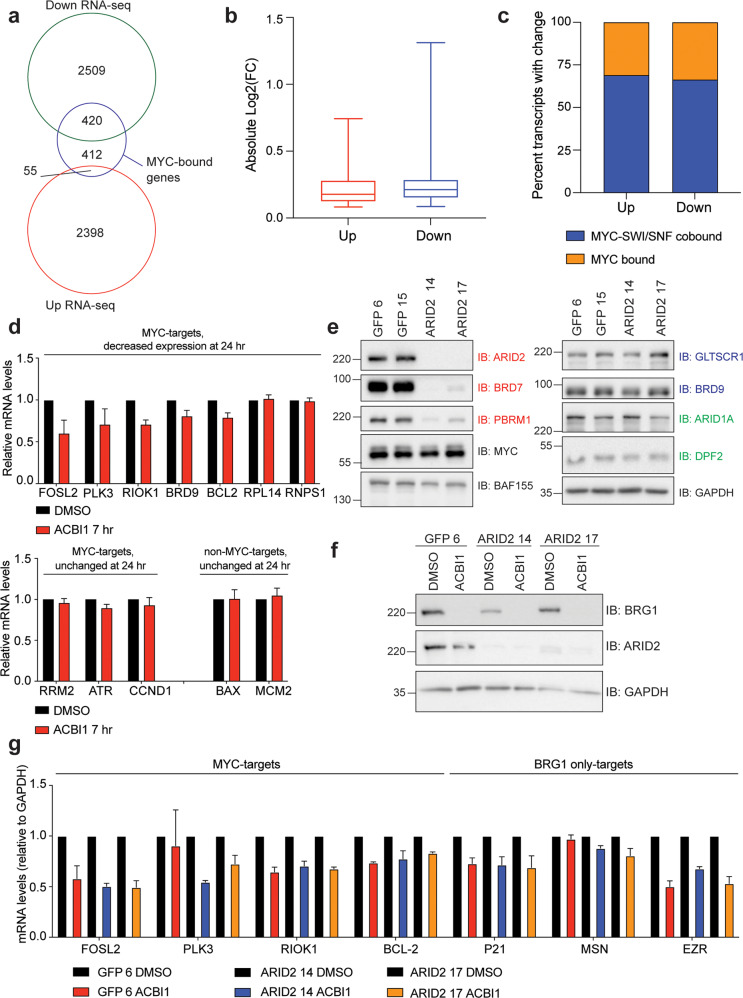
Loss of BRG1 impacts MYC target gene expression. **a** Venn diagram comparing transcript changes (Fig. 2a) to MYC-bound genes as determined by ChIP-seq [7]. **b** Box-and-whisker plot showing the absolute log2FC change for the 420 MYC-bound genes with decreased expression or the 55 MYC-bound genes with increased expression identified in **a**. Plot shows the 25th to 75th percentile, middle line marks the median with whiskers extending from minimum to maximum point. **c** Stacked bar graph showing the percentage of all MYC-bound genes with a transcript change in **a** that are also bound by BRG1 and BAF155 (“SWI/SNF”, [15]). **d** mRNA analysis of indicated genes following treatment of G401 cells for 7 h with ACBI1 or DMSO control as determined by RT-QPCR analysis. GAPDH is used as a reference gene for normalization (*n* = 3 biological replicates, error bars are standard error). **e** ARID2 was removed in G401 cells using CRISPR/Cas9 technology and clones validated for removal of ARID2 by western blot. ARID2 clone 14 and 17 are compared to two control clonal lines in which CRISPR was performed using a guide targeting the green fluorescent protein (GFP) gene. ARID2 loss causes specific reduction in pBAF complex subunit members PBRM1 and BRD7. GAPDH is used as a loading control. Red text denote pBAF-specific subunits, blue text are ncBAF specific subunits, and green text is cBAF specific subunits. **f** Indicated cell lines were treated for 24 h with 250 nM ACBI1 or DMSO control and protein lysates probed for BRG1 depletion. **g** mRNA analysis of MYC- or BRG1-only targets following treatment of indicated cell lines for 24 h with ACBI1 or DMSO control as determined by RT-QPCR analysis. GAPDH is used as a reference gene for normalization (*n* = 3 biological replicates, error bars are standard error).

In order to determine the extent of gene expression changes resulting from loss of rSWI/SNF function, we performed RNA-seq on G401 cells treated with 250 nM ACBI1 compared to DMSO control at 24 h post-treatment (Supplementary Fig. 1a), a timepoint at which no apparent growth changes are present (Fig. 1c). Overall, there are ~5000 gene expression changes (Fig. 2a, Supplementary Table 1) showing diverse responses in either direction (Fig. 2b). About 70–75% of genes that changed in expression (FDR < 0.05) are bound by BRG1 as determined by comparing differentially expressed genes to a publicly available BRG1 ChIP-seq dataset that previously determined BRG1-binding sites in untreated G401 cells (GSE90634, [4]) (Fig. 2c). In addition, BRG1-bound genes show a lower fold change in response when compared to non-BRG1-bound genes (Fig. 2d). Gene ontology (GO) enrichment analysis (https://david.ncifcrf.gov/) of genes with increased transcript levels shows multiple unique gene categories seemingly linked to developmental processes (Supplementary Fig. 1b), but gene set enrichment analysis (GSEA) reveals that genes increased in expression are positively enriched among only two MSigDB hallmark gene lists (FDR < 0.05) (Supplementary Table 2), one of which is”KRAS_signaling_down” (Fig. 2e). In contrast, GO analysis of genes with decreased expression shows strong enrichments among several essential biological functions such as ribosome biogenesis, cell cycle, and translation (Fig. 2f). GSEA also shows significant enrichments of decreased transcripts among 22 hallmark datasets (Supplementary Table 2), including both hallmark MYC target gene lists (Fig. 3g), E2F2 targets, unfolded protein response, MTORC1 signaling, and hypoxia (Supplementary Fig. 1c). We conclude based on these data that ACBI1 treatment predominately impacts the expression of BRG1-bound genes and that the primary role of rSWI/SNF in G401 cells is to facilitate the expression of genes linked to several essential biological functions.

### BRG1 regulates MYC target gene expression

Recently, we discovered that the oncogene MYC acts as a driver of MRT processes [7, 15] and that MYC can interact physically and colocalize on chromatin with rSWI/SNF subunits [15], suggesting that SNF5-null SWI/SNF complexes may serve to facilitate MYC-dependent gene expression in MRT. Given that hallmark MYC targets were the most significantly enriched GSEA result (Fig. 2g, Supplementary Table 2), we compared genes that we previously identified as MYC-bound by ChIP-seq in G401 cells [7] to all ACBI1 RNA-seq changes. Strikingly, over 50% of all MYC-bound genes experience a change in expression following ACBI1 treatment, with almost all changes being in the decreased direction (Fig. 3a). Genes that were decreased also have a larger spread in magnitude of response than those that increase in expression (Fig. 3b). Further comparison of identified MYC-SWI/SNF co-bound genes [15] to transcript changes shows that of the MYC-bound genes that respond to ACBI1 treatment, over 60% of those genes also show SWI/SNF co-binding (Fig. 3c), which is consistent with ACBI1 treatment having a bias towards impacting BRG1-bound genes (Fig. 2c). mRNA analysis at 7 h post-ACBI1 treatment reveals over half of selected MYC-bound genes that were decreased in expression at 24 h as determined by RNA-seq also show a decrease at this timepoint, with the exception of *RPL14* and *RNPS1* (Fig. 3d, top). Other known MYC-bound genes that were not identified as differentially expressed in the RNA-seq also trend towards decreased expression, which appears specific because non-MYC-bound targets analyzed were unaffected (Fig. 3d, bottom). These data reveal that ACBI1 results in a rapid and selective change in the expression of MYC-bound targets.

Because ACBI1 depletes both BRG1 and PBRM1 (Fig. 1a, b), we sought to determine which subunit plays the main role in the inhibition of MYC target gene expression by reducing pBAF selectively by targeting the pBAF-specific subunit ARID2 by CRISPR. In ARID2 CRISPR clones, reduction in ARID2 protein results in a corresponding loss in PBRM1 and BRD7 protein levels (Fig. 3e), and clones show reduced cell proliferation and anchorage-independent growth when compared to a GFP control clone (Supplementary Fig. 2a, b). Pan-SWI/SNF subunits BAF155 (Fig. 3e) and BRG1 (Fig. 3f) are also reduced modestly, but ncBAF and cBAF subunit expression is unaffected (Fig. 3e), indicating that targeting ARID2 by CRISPR can selectively reduce the pBAF complex specifically. With pBAF—and PBRM1—no longer able to contribute to the cellular response, two ARID2 clones and one control clone (GFP) were treated with ACBI1 or DMSO control (Fig. 3f), followed by mRNA analysis. A comparison of transcript levels across all three cell lines indicates that both MYC- and BRG1-only targets are decreased in response to ACBI1 (Fig. 3g), indicating that BRG1 rather than PBRM1 is the major contributor to modulating MYC target gene expression in G401 cells as well as many other gene expression changes observed.

### BRG1 and MYC can control distinct gene expression programs

**Fig. 4:**
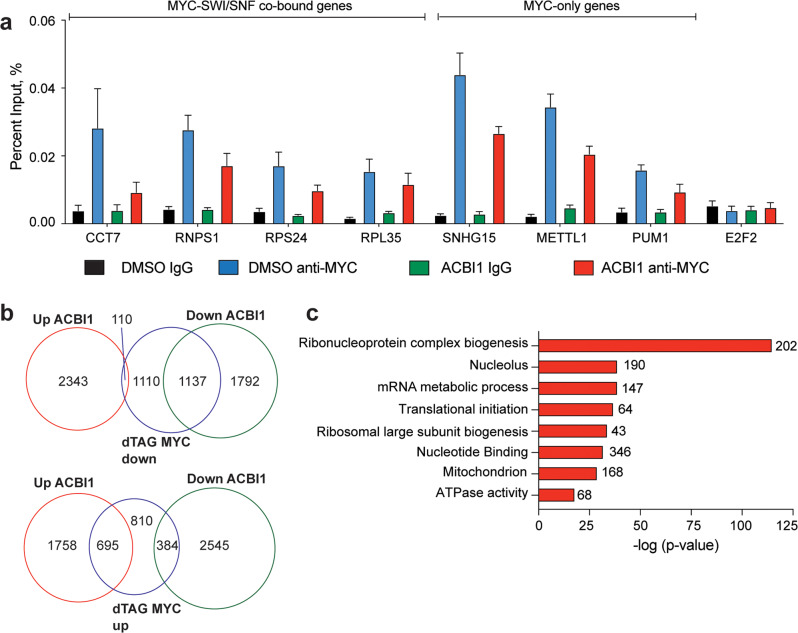
Removal of BRG1 compared to removal of MYC. **a** Chromatin immunoprecipitation followed by QPCR for various MYC-bound loci after ACBI1 treatment for 24 h. *E2F2* serves as a negative control for binding (*n* = 4 biological replicates, error bars are standard error). **b** Venn diagram comparing all significant transcript changes that result from ACBI1 treatment to decreased (top) or increased (bottom) gene expression changes that occur after removal of MYC using the dTAG approach [15]. **c** GO analysis of 1137 genes that are commonly decreased in expression across dTAG MYC and ACBI1 samples. Number of genes in each term are shown next to the bar.

Next, we sought to determine how BRG1 loss can lead to impaired MYC target gene expression. One possibility is that BRG1 can influence the ability of MYC to bind chromatin, which has been observed for SNF5 [7, 19]. To test this, we performed chromatin immunoprecipitation followed by quantitative polymerase chain reaction (QPCR) analysis. Over all loci examined, ACBI1 treatment caused a modest global reduction in MYC binding to chromatin but this effect was not specific to whether SWI/SNF is co-bound (Fig. 4a) and was not predictive of a change in transcript levels (Supplementary Table 1), suggesting that BRG1 loss may globally impact MYC binding to chromatin but that this does not correlate with transcript changes. However, because of the impact on MYC binding we observed, we decided to compare transcriptome changes from ACBI1 treatment to those we recently published in which we removed MYC in G401 cells through the dTAG approach [15, 20]. A comparison of these two RNA-seq datasets reveals that about half of genes with a decreased expression upon depletion of MYC are also decreased by the removal of BRG1 (Fig. 4b, top) while only ~37% of genes with increased expression in response to depletion of MYC are changed in the same direction when BRG1 is depleted (Fig. 4b, bottom). This suggests that while there are common biological processes controlled by MYC and BRG1 in MRT, each results in distinct changes to the transcriptome as well. GO analysis of these commonly downregulated genes highlights ribosome biogenesis as the top enriched biological process, along with nucleolar genes, ribosome protein genes, and mitochondrial genes (Fig. 4c). These data indicate that MYC and rSWI/SNF converge on genes that are required for protein synthesis and in particular, ribosomal function, which has been shown, at least for MYC, to be tied to MYC co-factor interactions [21, 22].

### ACBI1 treatment reduces chromatin accessibility at enhancers linked to signaling and migration genes

**Fig. 5:**
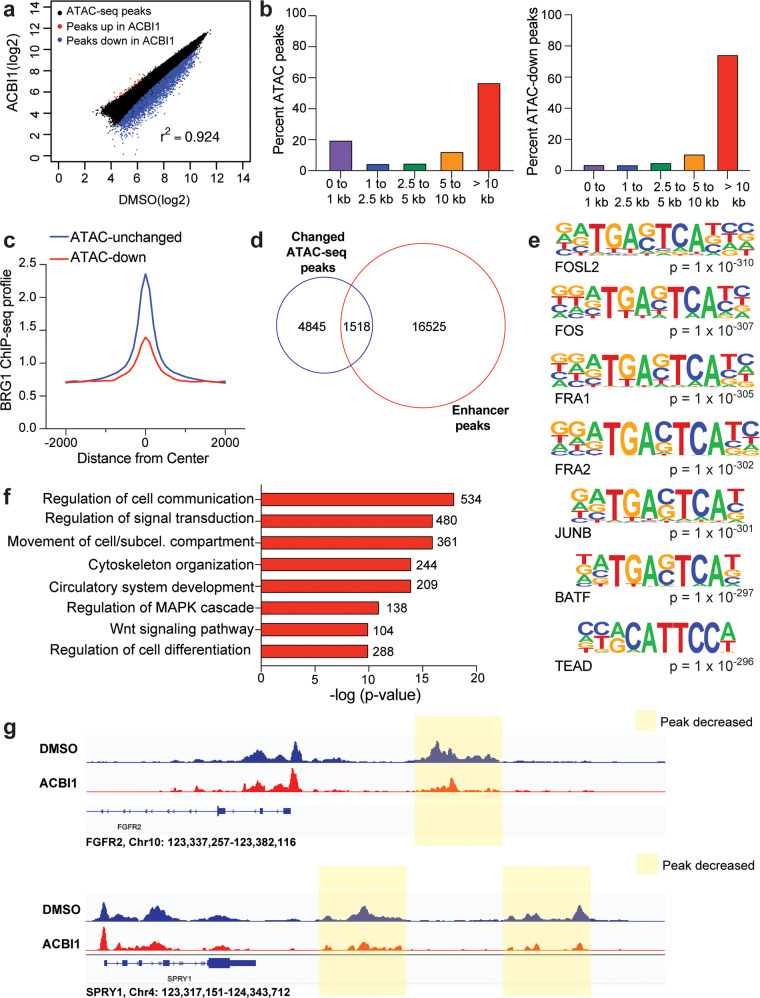
BRG1 regulates chromatin accessibility of genes associated with cancer hallmarks. **a** Scatterplot showing log_2_-fold changes of ATAC-peaks across DMSO vs. ACBI1. ATAC-peaks that are decreased upon ACBI1 treatment are shown in blue, while those increased are shown in red. **b** All ATAC-peaks (left) or ATAC-peaks that were significantly decreased (right, FDR < 0.05) were annotated to determine the distance of peak to the nearest transcription start site (TSS). Percent of ATAC-peaks falling into each bin are shown as bars within each graph. **c** Average normalized BRG1 ChIP-seq signal [4] at ATAC-sites which were unchanged in peak intensity or those with a decrease in ATAC-peak intensity are shown. **d** Venn diagram showing overlap between significantly changed ATAC-peaks (FDR < 0.05) and enhancer peaks in G401 cells. Enhancer peaks were identified as previously described [15]. **e** Top seven known motifs identified for decreased ATAC-peaks. **f** Decreased ATAC-peaks (FDR < 0.05, fold change < −1.5) were annotated to their nearest gene and GO analysis performed on all unique genes. Representative categories are shown, number of genes in each term are shown next to the bar. **g** IGV screenshot example of ATAC-sites that showed decreased peak intensity upon ACBI1 treatment. Nearest gene that sites were annotated to is shown to compare promotor-proximal peaks to promoter-distal sites. Peaks shaded in yellow are also identified as enhancers in **d**.

While rSWI/SNF has been shown to generally maintain gene expression in MRT [5, 6], its ability to actively remodel chromatin—and whether this retained function impacts gene expression—has not yet been determined. To test this, we treated G401 cells with ACBI1 for 24 h and performed the assay for transposase accessible chromatin coupled to next-generation sequencing (ATAC-seq) [23, 24]. Overall, we detected about 80,000 ATAC-peaks across all samples, with ~6000 peaks decreased in intensity and only ~240 peaks increased in intensity (Fig. 5a, Supplementary Fig. 3a, b), indicating that rSWI/SNF retains the ability to act as a chromatin remodeling complex in MRT cells. ATAC-peaks that are decreased in intensity showed a larger range of responses than the small number of peaks that increase (Supplementary Fig. 3c). Because decreased ATAC-peaks were the predominant change detected, we focused our detailed analysis on sites in which chromatin accessibility was reduced. When we annotated identified ATAC-peaks across all samples to determine their distance from the nearest transcription start site (TSS), we saw the majority of open chromatin sites detected fell into two groups, either proximal (<1 kb) or distal (>10 kb) from the nearest annotated TSS region (Fig. 5b, left). However, the ATAC-peaks that decreased in intensity are overwhelmingly TSS-distal (Fig. 5b, right) in comparison, and show lower BRG1 signal than ATAC-peaks that were unchanged (Fig. 5c), suggesting that ACBI1 treatment at least at this timepoint preferentially impacts TSS-distal regions with lowest BRG1 chromatin binding. Previously, TSS-distal enhancers were proposed as a driving mechanism for MRT [3] and therefore we compared changed ATAC-peaks with enhancer peaks called based on the presence of histone H3 lysine 27 acetylation (H3K27ac) without histone H3 lysine 4 tri-methylation (H3K4me3) [15] to determine how many reduced ATAC-peaks occur at histone marked enhancers. This analysis detects 1518 enhancer sites in which BRG1 depletion reduces chromatin accessibility—about 24% of all changed ATAC-peaks (Fig. 5d, Supplementary Table 3). Interestingly, motif analysis performed on decreased ATAC-peaks shows enrichment of the AP-1 transcription factor binding motif (Fig. 5e, Supplementary Table 4), a transcription factor that has been shown to work with SWI/SNF in enhancer selection and regulation of cell differentiation gene expression [7, 25, 26]. However, upon annotating decreased ATAC-peaks to their nearest gene and performing a GO analysis on those genes, we discovered that in contrast to differentiation and development genes, the genes with decreased ATAC-peak intensity were linked to several cancer hallmarks, including signaling, migration, and angiogenesis (Fig. 5f, g) in line with the role of AP-1 acting as a pro-oncogenic factor [27]. This was also evident when we performed a GO analysis on enhancers that have decreased ATAC-peak intensity (Supplementary Fig. 3d), suggesting that in addition to influencing MYC, rSWI/SNF maintains chromatin accessibility at enhancers whose function is to regulate genes involved in pro-tumorigenic processes.

### BRG1 regulates oncogenic gene expression through multiple mechanisms

**Fig. 6:**
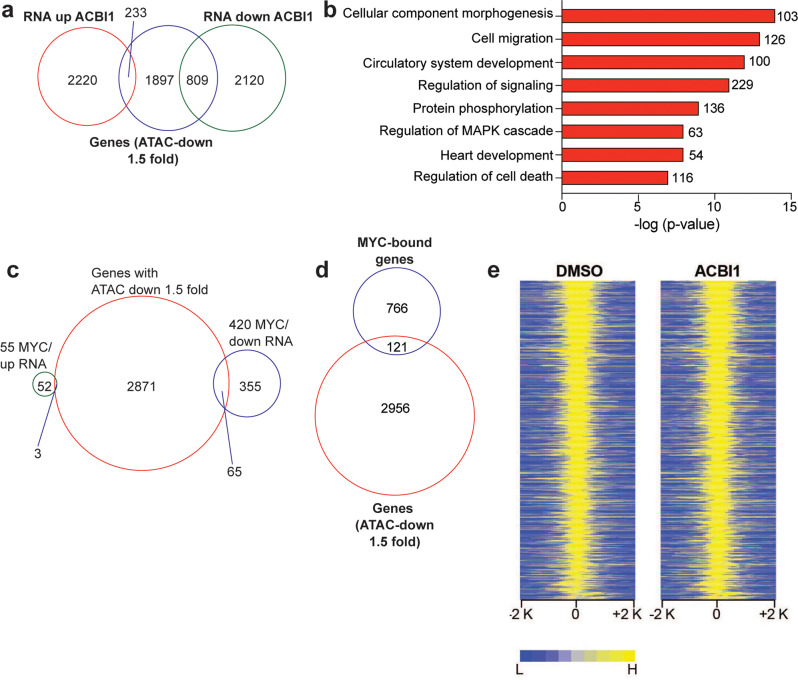
SWI/SNF controls multiple oncogenic gene expression programs through TSS-proximal and TSS-distal mechanisms. **a** Venn diagram showing genes associated with decreased ATAC-seq peak intensity (fold change < −1.5) and their overlap with genes that are increased or decreased in the RNA-seq analysis. **b** GO analysis of 809 genes in **a** that have decreased chromatin accessibility and a subsequent decrease in expression. **c** Venn diagram showing genes associated with decreased ATAC-seq peak intensity (fold change < −1.5) compared to MYC target genes that had changed gene expression in Fig. 3a. **d** Venn diagram showing genes associated with decreased ATAC-seq peak intensity (fold change < −1.5) compared to all MYC-bound genes in G401 cells. **e** Normalized ATAC-peak intensities for DMSO and ACBI1 samples are shown for all MYC-bound genes. ACBI1 peaks are ranked against those in DMSO sample.

Our ATAC-seq analysis suggests that rSWI/SNF complexes retain some chromatin remodeling function when SNF5 is not present and that most of their action at TSS-distal sites as a remodeler is separable from their action at TSS-proximal sites where MYC would be located. Therefore, to gain a deeper understanding of how chromatin remodeling changes relate to gene expression differences, we compared the ATAC-seq results to RNA-seq changes. First, we compared genes associated with the largest decreases in ATAC-peak intensity (fold change < −1.5) to genes that either increased or decreased in expression by RNA-seq. From this analysis 809 genes were identified that show decreased chromatin accessibility with a subsequent decrease in gene expression (Fig. 6a). Again, hallmark cancer processes emerged from a GO analysis of these genes, including signaling, migration, and angiogenesis (Fig. 6b). In comparison, MYC target genes that are differentially expressed by ACBI1 treatment show little overlap with genes having decreased ATAC-seq peaks (Fig. 6c). Even if all identified MYC-bound genes are compared to genes with ATAC-peak changes, there is only ~14% of sites overlap with changes in accessibility (Fig. 6d). Furthermore, heatmaps of ATAC-peak intensities for all MYC-bound genes show little difference across DMSO and ACBI1 samples (Fig. 6e). This suggests that BRG1 does not affect MYC target gene expression through any conspicuous chromatin remodeling. Taken together, this is compelling evidence for rSWI/SNF complexes having roles at TSS-proximal and TSS-distal sites to control well-known oncogenic processes and furthermore suggests that there are additional oncogenic processes that rSWI/SNF can regulate.

## Discussion

It is clear that SNF5 loss leads to a diverse set of changes in cellular function resulting in rhabdoid tumor formation [3–7, 25]. It is also evident that rSWI/SNF complexes remaining after SNF5 loss can maintain the oncogenic state, suggesting that rSWI/SNF may regulate known oncogenic processes and may do so through a variety of mechanisms given the breadth of the SWI/SNF complex types that have been characterized [5, 28]. Recently, we found that a major oncogene in cancer, MYC, is deregulated in MRT [7] and that rSWI/SNF subunits colocalize extensively with MYC on chromatin [15], providing the first links between rSWI/SNF complexes and oncogene function. Given that one of the normal functions of SNF5 within SWI/SNF is to antagonize MYC function [7, 19], we proposed originally that SNF5-null rSWI/SNF may be able to facilitate MYC target gene expression. In this study, we challenge the role of rSWI/SNF in impacting MYC target gene expression and uncover new mechanisms of oncogenesis that were not already known by using a recently developed selective PROTAC degrader called ACBI1 that degrades BRG1, so that we could eliminate full SWI/SNF function rather than focusing on any single SWI/SNF complex.

Targeting SWI/SNF complexes as we did here with a subunit-specific chemical degrader has readily gained traction as a promising means to treat cancers that depend on aberrant SWI/SNF function [29]. In addition to their therapeutic potential, PROTACs are very useful as biological tools because they allow rapid depletion and examination of biological responses, permitting acute changes to be detected [20] as we saw in our study. Importantly, during the development of ACBI1, an unbiased quantitative tandem mass tag labeling and proteomics assay was performed which showed ACBI1 treatment caused minimal decreases in any proteins other than BRG1, BRM, and PBRM1. And ACBI1 treatment only impacted cell growth of cancer cell lines known to depend on BRG1/BRM function—an effect that could be blocked by addition of a competing bromodomain ligand—demonstrating notable specificity and selectivity in the action of ACBI1 [16]. Subsequent analyses using this commercially available degrader as both a biological tool compound [18] and small molecule therapeutic [17] have been reported, expanding the use of this selective degrader as a means to inactivate SWI/SNF function rapidly. This well-characterized molecule was key to uncovering the novel findings in this study as chemical degradation of BRG1 allowed for analysis of rSWI/SNF function at much earlier timepoints than previously used when targeting rSWI/SNF [5, 6, 10].

Using 250 nM ACBI1 treatment, gene expression changes occur in as little as 24 h, which precedes overt cellular changes (Fig. 1c), and shows that removal of BRG1 does impact both expression of MYC target gene signatures (Fig. 2g, Supplementary Table 1) and MYC-bound targets we previously identified using ChIP-seq analysis (Fig. 3a). The overwhelming majority of changes to MYC targets was decreased gene expression, which occurred in as little as 7 h to select MYC target genes (Fig. 3d) and is specifically due to BRG1 and not the pBAF-specific subunit, PBRM1 (Fig. 3g), a subunit also degraded by ACBI1 (Fig. 1a, b). Overall, these data provide strong evidence that rSWI/SNF can facilitate MYC target gene expression. As the removal of pBAF did not affect ACBI1-induced gene expression changes (Fig. 3g), it is likely that ncBAF or cBAF SWI/SNF complexes are involved. Given that ncBAF shows promoter-proximal binding [5] like MYC [15] while cBAF is typically thought to be associated with promoter-distal enhancers [5], it may be that ncBAF is the specific complex that facilitates MYC target gene expression.

One unique function that SWI/SNF complexes have is the ability to remodel chromatin, although direct evidence for the capacity of rSWI/SNF to perform this function in MRT has not been provided until this study. Remarkably, ACBI1 treatment leads to thousands of open chromatin sites being reduced and almost all changes occur at TSS-distal regions in the MRT genome (Fig. 5b), indicating that rSWI/SNF actively maintains chromatin accessibility of specific genomic sites. The connection between TSS-distal regions and rSWI/SNF was made first by Roberts and colleagues [3], who identified enhancers that retain binding by rSWI/SNF subunits. A comparison of enhancers called based on differentiating histone marks reveals that about 25% of the sites with impacted chromatin accessibility in this study were indeed enhancers (Fig. 5d), suggesting that some enhancers in MRT are kept active at least in part by active remodeling at these locations.

To uncover more mechanistic insight into regions in which rSWI/SNF controls the open chromatin state, we performed motif analysis on decreased ATAC-peaks. Strikingly, AP-1 transcription factor motifs were among the most enriched motifs present (Fig. 5e, Supplementary Table 4), occurring in around 12–25% of peaks that had a decrease in open chromatin (Supplementary Table 4). AP-1 is a transcription factor that contains dimers of the Jun and Fos proteins with known pro-oncogenic and anti-oncogenic roles [27]. As it pertains to normal SWI/SNF and AP-1 interactions, their relationship has been increasingly linked to anti-oncogenic processes [7, 25, 30, 31] where it is thought that AP-1 and SWI/SNF interact through the BAF60a SWI/SNF subunit [26, 32] to allow for enhancer selection and activation of cell fate and differentiation gene expression programs [26]. However, in our study the sites with reduced chromatin accessibility are not associated with cell fate and differentiation genes but rather pro-oncogenic gene categories such as migration, signal transduction, and angiogenesis. This discovery suggests that in the absence of SNF5 in MRT, the selection of enhancers by SWI/SNF is impaired. We suspect this is due to the loss of SNF5 to target normal SWI/SNF complexes (and potentially AP-1) to enhancers linked to differentiation and development [3, 7, 25, 33]. Importantly, the cancer hallmark genes we identified in the ATAC-seq analysis are also present in human and mouse studies of rhabdoid tumors. For example, upregulated signaling pathways are activated in mouse models of SNF5 loss and rhabdoid patient samples [34, 35], while invasion/motility genes are also increased in patient-derived xenografts [36], suggesting the possibility that altered AP-1 function may be involved driving the rhabdoid tumor state.

Many of the genes associated with reduced chromatin accessibility when BRG1 is removed also have reduced gene expression at the same timepoint (Fig. 6a, b) showing that accessible chromatin is important for proper gene expression in MRT. However, decreased MYC target gene expression cannot be explained by overt changes to the open chromatin state (Fig. 6d, e), indicating that the ability of rSWI/SNF to impact MYC target gene expression may be through a mechanism not fully dependent on chromatin remodeling. Currently, we do not know the mechanism by which rSWI/SNF causes reduced MYC target gene expression, but we do not think it is due to any impact on MYC chromatin binding (Fig. 4a) as changes in MYC binding do not correlate with changes in gene expression. It is possible that rSWI/SNF functions in other aspects of MYC-dependent transcription, including RNA polymerase recruitment or activation, serving as a boundary/insulator for MYC target gene expression, recruitment of an important co-factor or co-regulator, or an otherwise unidentified process. Further investigation will be required to fully elucidate these mechanisms.

Overall, our data expose the role of rSWI/SNF in controlling MYC function and suggest that in addition to MYC, AP-1 is involved in the maintenance of the rhabdoid tumor state. Considering these data, we propose a revised model for thinking about how rSWI/SNF complexes maintain aberrant transcriptional programs in rhabdoid tumors: SNF5 normally functions to both temper MYC binding to chromatin [7, 19] and activate enhancers controlling differentiation and development genes [3, 4], the combination of which allows for the integration of signals dictating normal cell growth during development. However, upon SNF5 loss these critical functions are absent, collapsing the cell to a primordial ground state that becomes locked into place due to continued support of pro-tumorigenic gene expression by rSWI/SNF complexes. In this view, oncogenes and rSWI/SNF together drive rhabdoid tumor processes and it is possible that inhibition of either driver may ultimately inhibit cancer function. Taken together, the evidence provided in this study may predict specific oncogenic pathways that become activated when SWI/SNF is mutated, which occurs in ~20% of all cancers [37].

## Materials and methods

### Cell culture and ACBI1 treatment

G401 cells were obtained from ATCC and HEK293T cells are in-house stocks. HEK293T and G401 cells, including G401 CRISPR clones, were maintained in DMEM supplemented with 1% Penicillin/Streptomycin and 10% fetal bovine serum (FBS). ACBI1 (MedChemExpress, 2375564-55-7) was prepared in dimethyl sulfoxide (DMSO) upon arrival. ACBI1 treatments were performed by incubating indicated numbers of cells in media containing 250 nM ACBI1 or matched DMSO for the duration of the treatment. Cell lines were tested for mycoplasma contamination using a Mycoplasma PCR Detection Kit (MP Biomedicals) following genomic DNA extraction with the Purelink Genomic DNA kit (Thermo Scientific).

### RNA-seq and mRNA analysis

1–2 × 10^6^ of indicated cells were plated with media containing 250 nM ACBI1 or DMSO. After 24 h, cells were collected in Trizol, and RNA was extracted using the Direct-zol RNA mini-prep kit (Zymo Research). For RNA-seq, 1–2 μg was submitted to Vanderbilt Technologies for Advanced Genomics (VANTAGE) core at Vanderbilt University Medical Center for ribosomal RNA depletion and library generation. 150 bp paired-end reads were obtained on an Illumina NovaSeq6000 instrument. For RT-qPCR analysis, purified RNA was reverse transcribed to cDNA using MulV reverse transcriptase (Promega). cDNA was analyzed using the AriaMx Real-Time PCR Machine (Agilent) and *GAPDH* was used as a reference gene. All primer sequences used in this study are in Supplementary Table 5. Three biological replicates were used for each assay.

### ATAC-seq and analysis

1 × 10^6^ cells were treated with 250 nM ACBI1 or DMSO control for 24 h. For each ATAC-seq sample, cells were counted, and 75,000 cells harvested and lysed in the ATAC Lysis buffer supplied by Active Motif ATAC-Seq kit (53150). The remaining steps including the transposase reaction, library generation, and library purification were performed according to the manufacturer’s instructions except for the final step in which libraries were cleaned up using a PCR purification kit (Qiagen) instead of SPRI beads. Libraries were submitted to VANTAGE at Vanderbilt University Medical Center and sequenced using their ATAC-seq sequencing protocol on an Illumina NovaSeq6000 to obtain 150 bp paired-end reads. Three biological replicates were used for ATAC-seq.

### ATAC-seq analysis

After adaptor trimming by Cutadapt [38] (cutadapt -j 2 -O 1 -n 3 -q 20 -a CTGTCTCTTATA -A CTGTCTCTTATA -m 30 --trim-n), ATAC-seq reads were aligned to the human genome hg19 using Bowtie2 (bowtie2 -p 8 -X 2000 --no-mixed --no-discordant) [39] [bowtie2 ref]. Peaks were called using MACS2 with q-value of 0.05 [40]. Differential enriched peaks were identified using DiffBind [41]. Peaks present in at least two replicates per condition were included, and peaks identified across conditions were combined into a final peak set and ATAC-seq read counts were calculated for the final peak set.

### RNA-seq analysis

After adapter trimming by Cutadapt [38], (cutadapt -j 2 -q 20 -a AGATCGGAAGAGCACACGTC -A AGATCGGAAGAGCGTCGTGT -m 30 -n 2) reads were aligned to the hg19 human genome using STAR [42] and quantified by featureCounts [43]. Differential analysis was performed using DESeq2 [44], which determined the Wald test p-values, log2 fold changes, and adjusted *p*-value (FDR) by the Benjamini–Hochberg procedure. The significantly changed genes were assessed with a FDR < 0.05. Three biological replicates were used for RNA-seq.

## Supplementary information

Supplementary Material

Supplementary Table 1

Supplementary Table 2

Supplementary Table 3

Supplementary Table 4

Supplementary Table 5

## Data Availability

Sequencing data generated in this study are deposited on GEO with the accession number GSE198156. MYC- and BRG1-binding sites in untreated G401 cells used in this study were previously identified using ChIP-seq analysis [4, 7, 15] and can be found under the accession numbers GSE109310 and GSE90634, respectively. Additional data may be provided upon request.
