# Supplementary material for: The SWI/SNF ATPase BRG1 facilitates multiple pro-tumorigenic gene expression programs in SMARCB1-deficient cancer cells
Source: Oncogenesis. 2022 Jun 1;11(1):30. doi: 10.1038/s41389-022-00406-6 (PMC9160003; doi:10.1038/s41389-022-00406-6)
Supplement: Supplementary file 1 — Supplementary Material [file 41389_2022_406_MOESM1_ESM.pdf]

Supplementary Material

The SWI/SNF ATPase BRG1 facilitates multiple pro-tumorigenic gene expression programs in SMARCB1-deficient cancer cells

Moe, K.C. et al.

Included figures:

Supplementary Figure 1. ACBI1 treatment causes decreased gene expression changes of genes linked to important biological functions.

Supplementary Figure 2. Removal of pBAF complex impairs anchorage-independent growth.

Supplementary Figure 3. ACBI1 treatment causes a predominant loss in chromatin accessibility at sites linked to signaling, migration, and growth.

Excel files:

Supplementary Table 1. List of differentially expressed genes following treatment with ACBI1. Gene expression changes that result from G401 cells treated with 250 nM ACBI1 or DMSO control for 24 hr.

Supplementary Table 2. Gene set enrichment analysis (GSEA) report for ACBI1-induced gene expression changes ranked against MSigDB Hallmark gene sets. GSEA was performed using the list of differentially expressed genes from RNA-seq compared to MSigDB Hallmark gene sets.

Supplementary Table 3. Annotation of peaks that are identified as enhancer sites and have a corresponding change in chromatin accessibility. Annotation was performed on the ATAC-peaks that overlap with enhancer peaks described in Fig. 5d.

Supplementary Table 4. Known motif analysis of ATAC-seq peaks that were decreased in peak intensity following ACBI1 treatment. Motif analysis was performed on peaks that decreased in ATAC-seq peak intensity after ACBI1 treatment.

Supplementary Table 5. Primer sequences used in this study. A detailed list of primers used in this study and which application they were used for.

Extended Materials and Methods

Generation of CRISPR ARID2 clones. To target ARID2 using CRISPR, two guide sequences against ARID2 (AGCTGTGGCTTTGGATTGCC and TACCCGACACACTGTGTTGC) or a guide sequence targeting green fluorescent protein (GAGCTGGACGGCGACGTAAA) were inserted into the pSpCas9(BB)-2A-Puro (PX459) vector (1) following phosphorylation and annealing of oligos. These plasmids were transfected into G401 cells using Lipofectamine 3000 (*Life Technologies*) and after three days, cells were pulsed with 1 µg/ml puromycin for an additional three days. Clones were generated following a limited dilution assay and validated using antibodies against ARID2 using western blot. pSpCas9(BB)-2A-Puro (PX459) V2.0 was a gift from Feng Zhang (Addgene plasmid # 62988; http://n2t.net/addgene:62988; RRID: Addgene_62988)

Cell proliferation and cell cycle analysis. 5 x 10^5 of each indicated cell line were plated equally onto separate plates. At the end point, cells were counted using the ThermoFisher Countess II and total cell number calculated. For cell cycle analysis, 5 x 10^5 cells were plated in

media containing 250 nM ACBI1 or DMSO. After four days, 1 x 10^6 cells were collected and fixed in cold 70% ethanol and stored at -20°C overnight. Before staining, fixed cells were allowed to thaw at room temperature, then washed with 1X phosphate buffered saline (PBS). Cells were resuspended in propidium iodide (PI) staining solution (1X PBS + 10 μg/ml PI + 100 μg/ml RNAse A + 2 mM $MgCl_2$) and left to stain overnight at 4°C. Stained cells were filtered through a 35 μm nylon mesh cell strainer Falcon tube before being counted with on a Guava easyCyte Flow Cytometer instrument (Luminex). A minimum of 10,000 cells were counted for each sample, with single cells being selected based on forward and side scatter. For cell proliferation five biological replicates were performed and for cell cycle analysis four biological replicates were performed. All associated statistical comparison information can be found in the appropriate figure legend.

Western blotting and antibodies. 2 x 10^6 cells were treated with compounds for the indicated timepoint. DMSO was matched to the longest timepoint. Following treatment, cells were collected in cold lysis buffer (150 mM Tris, pH 8.0, 5 mM EDTA, 150 mM NaCl, 1% Triton X-100, supplemented with Roche protease inhibitor cocktail and PMSF), sonicated at 25% power for 15 seconds, and centrifuged to separate cellular debris. The BioRad Bradford assay was used to determine protein concentration. 10-30 μg of protein per sample was resolved by SDS-PAGE before being transferred to a PVDF membrane (PerkinElmer) and allowed to block in 5% milk made in TBS-T (50 mM Tris, pH 7.5, 0.1% Tween-20, 150 mM NaCl). Antibodies used for immunoblotting were as follows: ARID2 (Cell Signaling, D8D8U), BRD7 (Cell Signaling, D9K2T), BRG1 (Cell Signaling, D1Q7F), GAPDH-HRP (Cell Signaling, D16H11), c-MYC (Cell Signaling, E5Q6W), PBRM1 (Cell Signaling, E9X2Z). Bands were visualized using the Clarity ECL substrate (BioRad) on a Bio-Rad ChemiDoc MP instrument.

Chromatin immunoprecipitation. G401 cells were plated at 10 x 10^6 and treated with 250 nM ACBI1 or matched DMSO control for 24 hours. 1% formaldehyde was added to cells for 10 minutes and then reaction was quenched with 0.125 M glycine for an additional 10 minutes. Cells were washed in 1X PBS and pelleted by centrifugation. Nuclei extraction was performed in a nuclear lysis buffer (10 mM HEPES, pH 7.9, 0.4% NP-40, 10 mM KCl) on ice for 5 min before pelleting nuclei at 1500 rpm. Nuclei were then incubated in FALB buffer (50 mM HEPES, pH 7.5, 1 mM EDTA, 140 mM NaCl, 1% Triton) containing 1% SDS for a minimum of 15 minutes before chromatin was sheared using a Diagenode Bioruptor. Debris were cleared by centrifugation and chromatin frozen at -80°C. Immunoprecipitations were performed by diluting chromatin in FALB buffer without SDS and adding the following antibodies: 800 ng rabbit IgG control (Cell Signaling, 2729S) or 5 µl MYC (Abcam, Y69, 32072). Immunoprecipitated complexes were bound to protein A agarose (Roche) and washed extensively as previously described (2, 3) Following reversal of crosslinks, DNA was analyzed on an AriaMx Real-Time PCR Machine (Agilent) using primers listed in **Supplementary Table 5**. Four biological replicated were used for this assay.

References for Materials and Methods

1. Ran FA, Hsu PD, Wright J, Agarwala V, Scott DA, Zhang F. Genome engineering using the CRISPR-Cas9 system. Nat Protoc. 2013;8(11):2281-308.
2. Weissmiller AM, Wang J, Lorey SL, Howard GC, Martinez E, Liu Q, et al. Inhibition of MYC by the SMARCB1 tumor suppressor. Nat Commun. 2019;10(1):2014.
3. Woodley CM, Romer AS, Wang J, Guarnaccia AD, Elion DL, Maxwell JN, et al. Multiple interactions of the oncoprotein transcription factor MYC with the SWI/SNF chromatin remodeler. Oncogene. 2021;40(20):3593-609.

Supplementary Figure 1

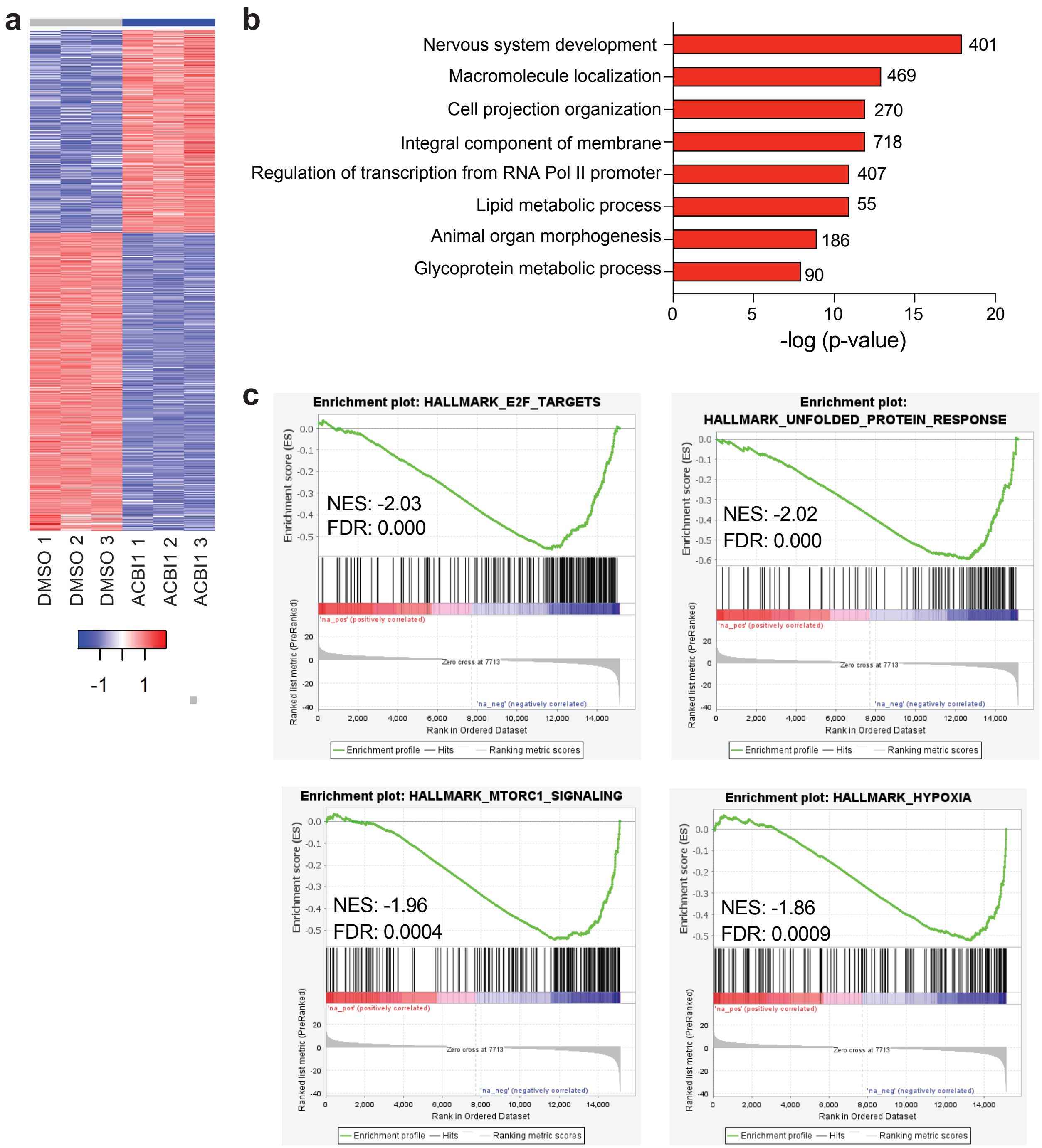

Supplementary Figure 1. ACBI1 treatment causes decreased gene expression changes of genes linked to important biological functions. (a) z-transformed heatmap showing all normalized read counts across each of the replicates used for RNA-seq analysis. (b) GO analysis of transcripts that are increased by ACBI1 treatment. Number of genes in each term are shown next to the bar. (g) GSEA analysis showing significant MSigDB hallmark lists that resulted from comparing all gene expression changes with ACBI1 treatment against the MSigDB hallmark data sets. Transcripts that are changed with ACBI1 treatment show a negative enrichment within the hallmark datasets.

Supplementary Figure 2

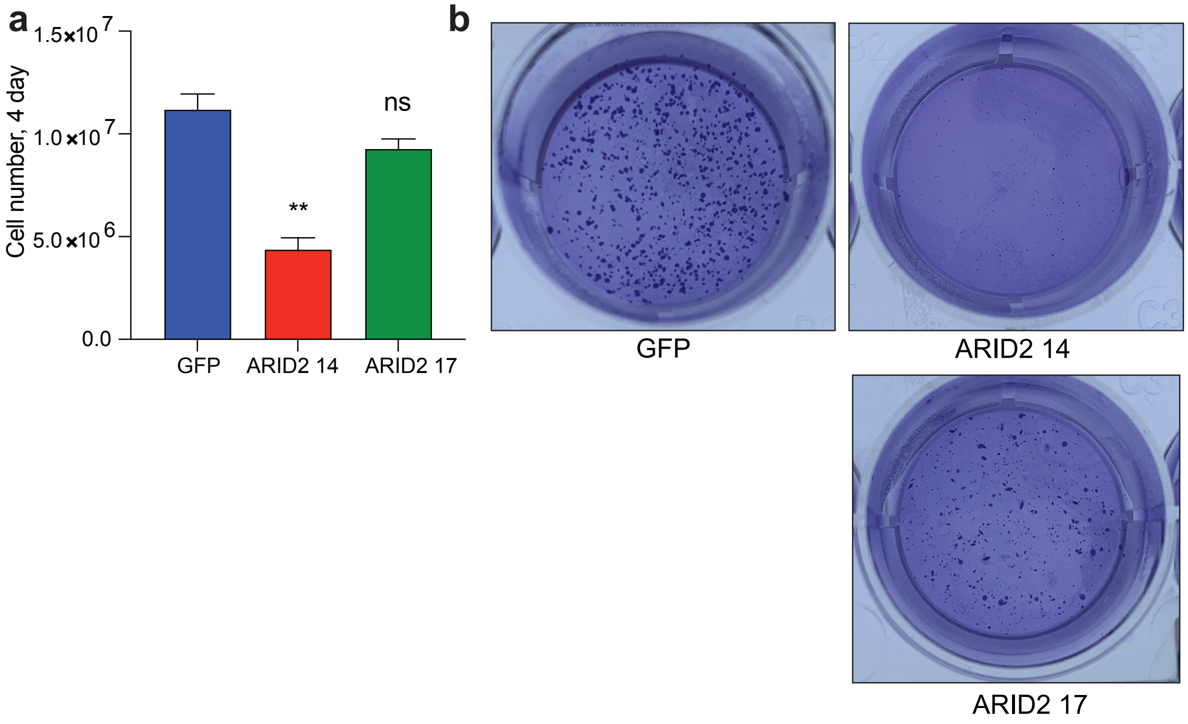

Supplementary Figure 2. Removal of pBAF complex impairs anchorage-independent growth. (a) G401 clones were plated equally and allowed to grow for four days before counting (n = 3 biological replicates, error bars are standard error, ns = not significant, P = 0.10 **P = 0.002 using unpaired t-test, two-tailed). (b) Soft agar assay for indicated clones was performed and representative images shown. Colonies were allowed to form for 14 days (n = 3 biological replicates).

Supplementary Figure 3

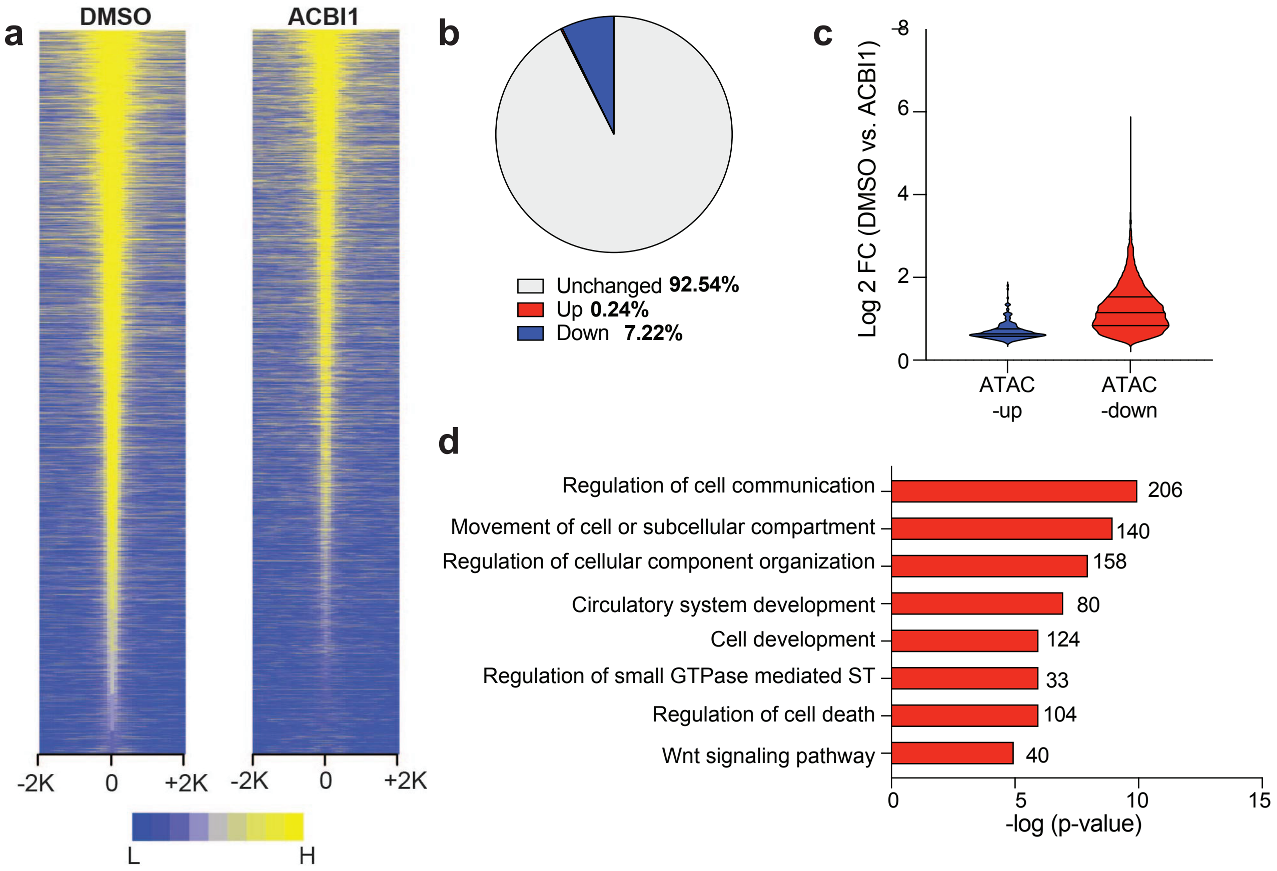

Supplementary Figure 3. ACBI1 treatment causes a predominant loss in chromatin accessibility at sites linked to signaling, migration, and growth. (a) Normalized ATAC-peak intensities for DMSO and ACBI1 samples are shown. ACBI1 peaks are ranked against those in DMSO sample. (b) Pie chart representing percentage of peaks that were decreased or increased in peak intensity, compared to all ATAC-peaks identified. (c) Violin plot of absolute log2FC changes for ATAC-peaks that were increased (ATAC-up) or decreased (ATAC-down) upon ACBI1 treatment. (d) 1518 overlapped peaks in Fig. 5d were annotated to their nearest gene and a GO analysis performed. Number of genes in each term are shown next to the bar. "ST" stands for "signal transduction".
